# Discrimination in the United States: Perspectives for the future

**DOI:** 10.1111/1475-6773.13218

**Published:** 2019-10-24

**Authors:** Robert J. Blendon, Logan S. Casey

**Affiliations:** ^1^ Department of Health Policy and Management Harvard T.H. Chan School of Public Health Boston Massachusetts

**Keywords:** Determinants of Health/Population Health/Socioeconomic Causes of Health, Racial/Ethnic Differences in Health and Health Care, Social Determinants of Health, Survey Research and Questionnaire Design

## Abstract

**Objective:**

To summarize findings from this Special Issue, which examine reported experiences of discrimination among six underrepresented groups in public opinion research—blacks, Latinos, Native Americans, Asian Americans, lesbian, gay, bisexual, transgender, or queer (LGBTQ) adults, and women.

**Data Source and Study Design:**

Data come from a nationally representative, probability‐based telephone survey of 3453 US adults, conducted January—April 2017.

**Methods:**

We calculated the percent of adults reporting discrimination in several domains, including health care.

**Principal Findings:**

In health care encounters, 32 percent of black adults reported discrimination, as did 23 percent of Native Americans, 20 percent of Latinos, 18 percent of women, 16 percent of LGBTQ adults, and 13 percent of Asian Americans. Significant shares also reported experiencing racial, gender, or LGBTQ identity‐based violence against themselves or family members, including 51 percent of LGBTQ adults, 42 percent of blacks, 38 percent of Native Americans, and 21 percent of women. At least one in seven blacks (22 percent), LGBTQ adults (18 percent), Latinos (17 percent), and Native Americans (15 percent) reported avoiding health care for themselves or family members over concerns of anticipated discrimination or unfair treatment.

**Conclusions:**

Taken together, this polling effort illustrates the significant and widespread level of discrimination against many groups in America today, as well as the complex manifestation of these experiences across different groups and different areas of life. While it is beyond the scope of these results to make specific recommendations for how to end discrimination in each area of life we studied, this Special Issue provides important evidence that more research and practice on discrimination are sorely needed in health services research.

## 1 INTRODUCTION

In 2014, a national coalition representing 200 US civil and human rights groups released a report, *Falling Further Behind: Combating Racial Discrimination in America*.^1^ This report detailed federal policy progress and opportunities to eliminate discrimination against minorities in key areas that affect their lives and health, including health care, criminal justice, education, employment, housing, and voting. Concurrently, in recent years public opinion polls have shown major divisions in public views about the existence and seriousness of racism, sexism, and other forms of discrimination in the United States.^2^, ^3^, ^4^, ^5^, ^6^, ^7^, ^8^ Recent polling has documented large gaps between whites and minorities over their general beliefs about racial/ethnic discrimination in the United States today, with similar divisions in beliefs about whether discrimination exists against women and lesbian, gay, bisexual, transgender, or queer (LGBTQ) adults.^2^, ^3^ The 2017 *Discrimination in the United States* polling effort at the heart of this special issue was conducted in the midst of this national debate to answer the question of how serious discrimination is in the lives of Americans across important policy domains and interpersonally, from the perspectives of four racial/ethnic minority groups—blacks, Latinos, Native Americans, and Asian Americans—as well as women and LGBTQ adults.

The question of whether these adults face serious discrimination, and the divide in public opinion over its answer, are critical to policy actions to end discrimination in the United States, as well as the health outcomes of minority and marginalized populations. For example, the Trump administration has begun reversing several Obama‐era antidiscrimination policies, including policies on health care, college admissions, housing, and fair lending.^8^ This creates an uncertain future of whether and how federal policies may play a role in perpetuating or reducing bias against persons because of their gender, race, ethnicity, or sexual orientation. As Williams et al have shown in this Special Issue, major patterns of racism, sexism, and other discrimination can significantly harm the health and broader well‐being of impacted populations, adding even more importance to these questions.

The purpose of this polling effort was to bring a public health perspective to the complexity and pervasiveness of discrimination in the United States today, in both health and nonhealth areas. We believe new research on discrimination is essential for the field of health services research, as social determinants of health can have important long‐term impacts on population health and well‐being.^9^, ^10^, ^11^, ^12^ Areas studied in this poll affect over a dozen areas of American life, including adults' health care, safety, housing, and opportunities in education and the workplace. This polling effort extends prior work in this area by: focusing on people's reports of their own direct life experiences with discrimination, rather than general perceptions of discrimination in the country broadly. It also simultaneously captures these reported life experiences among six underrepresented groups in public opinion research on discrimination—blacks, Latinos, Native Americans, Asian Americans, LGBTQ adults, and women. Because these groups are difficult to sample in telephone polling, their experiences often overlooked in overall results. We asked about experiences of discrimination among 3453 adults ages 18 and older, including nationally representative samples of each subgroup (see Table 1 for data on individual subgroup sample sizes). Discrimination was conceptualized as differential or unfair treatment of individuals based on self‐identified race, ethnicity, gender identity, or sexual orientation, whether by individuals or social institutions.^12^, ^13^, ^14^, ^15^

**Table 1:** Reported discrimination (weighted percent), by race/ethnicity and among women and LGBTQ adults^a^

		Whites	Blacks	Latinos	Native Americans	Asians	Women	LGBTQ Adults
		N = 902	N = 802	N = 803	N = 342	N = 500	N = 1596	N = 489
	Subject of Discrimination^b^	Weighted percent of respondents						
General belief that discrimination against [your racial/ethnic/gender/LGBTQ identity] exists today in the United States^c^	All adults	55	92	78	75	61	68	91
Going to a doctor or health clinic	You	5	32	20	23	13	18	16
Avoided doctor or health care because of concerns of discrimination/poor treatment	You or family member	3	22	17	15	9	9	18
Applying for jobs^d^	You	19	56	33	31	27	31	20
Interacting with police	You	10	50	27	29	18	15	16
Violence^e^	You or family member	13	42	20	38	10	21	51

a Non‐Hispanic whites, blacks, Latinos, Native Americans, Asian Americans, Women, and lesbian, gay, bisexual, transgender, or queer (LGBTQ) adults, ages 18+. Several questions were only asked among a randomized subsample of half of respondents within each category. Don't know/refused responses are included in the total n for each question but are not shown.

b Questions about you are personal experiences only; questions about you or family member ask if items have happened to you or a family member because you or they are [respondent's own racial/ethnic/gender/LGBTQ identity].

c All adults asked about discrimination against [respondent's own racial/ethnic/gender/LGBTQ identity] in America today. Question asked as “Generally speaking, do you believe there is or is not discrimination against [respondent's own race] in America today?”

d Jobs question only asked among respondents who have ever applied for a job.

e Question wording: “Do you believe that you or someone in your family has experienced violence because you or they are [respondent's own racial/ethnic/gender/LGBTQ identity].”

The papers in this Special Issue detail the widespread prevalence of reported discrimination by racial/ethnic minorities, women, and LGBTQ adults. Here, we briefly summarize key results in Table 1. In health care encounters, 32 percent of black adults reported discrimination, as did 23 percent of Native Americans, 20 percent of Latinos, 18 percent of women, 16 percent of LGBTQ adults, and 13 percent of Asian Americans. In addition, sizeable shares of adults reported avoiding health care for themselves or family members over concerns of anticipated discrimination or unfair treatment. For example, 22 percent of black adults reported avoiding health care for this reason, as did 18 percent of LGBTQ adults, 17 percent of Latinos, and 15 percent of Native Americans.

Adults from several minority or marginalized groups also reported experiencing racial, gender, or LGBTQ identity‐based violence against themselves or family members, including 51 percent of LGBTQ adults, 42 percent of black adults, and 38 percent of Native Americans. In addition, one‐quarter or more blacks, Latinos, and Native Americans reported discrimination in applying for jobs and police interactions.

Though some may expect that improving educational attainment or higher income may act as shields from discrimination, several papers in this Special Issue found that education and income are not protective against discrimination for several groups of inquiry (see Steelfisher et al, Findling et al, and Bleich et al). For several groups we studied, having greater education is associated with *higher* reported discrimination, whereas the reverse is true among whites.

In addition, some Americans face compounded discrimination in health care due to their race, ethnicity, gender, or sexual orientation. For example, in this Special Issue Steelfisher et al and Casey et al show greater reported discrimination among adults with intersectional minority/marginalized identities, such as for nonwhite LGBTQ women, who may face discrimination along multiple dimensions of their social identities.

Beyond lived experiences, adults from all surveyed groups overwhelmingly held general beliefs that discrimination against their own racial, ethnic, gender, or LGBTQ identity group exists in the United States today, with 92 percent of blacks, 91 percent of LGBTQ adults, 78 percent of Latinos, 75 percent of Native Americans, 68 percent of Women, 61 percent of Asians, and even 55 percent of whites saying discrimination exists against their group today.

Based on reports from individuals' own lives detailed in the papers in this Special Issue, we see a pattern of substantial reported institutional and individual discrimination against members of various minority and marginalized communities. These results extend prior evidence that despite public perceptions to the contrary, discrimination is a widespread and serious problem in America today, as experienced by several racial/ethnic minority groups, women, and LGBTQ adults.^2^, ^3^, ^7^ Even though these experiences are self‐reported, prior literature has shown that perceived, self‐reported discrimination is associated with worse health outcomes.^12^, ^14^, ^15^ Experiences of discrimination not only violate Americans' civil rights, but they exacerbate life opportunities and health disparities.

Eliminating discrimination will require leadership in multiple sectors of society, including government and health care. Of particular importance for our health care community, while financial barriers to health care are widely discussed and remain a significant obstacle in America, this survey also reveals that discrimination itself is a barrier to health care for many Americans. Thus, significant shares of Americans from different backgrounds avoid seeking health care due to anticipated discrimination and concerns that they or family members will experience unfair treatment because of their identity. This is extremely troubling in the post‐Affordable Care Act era. The extent of discrimination across groups when they seek medical care, based on their racial/ethnic backgrounds, gender identity, or sexual orientation, highlights how much work remains for equal treatment to become a reality. In addition, our findings in job applications, policing, and violence highlight similar problems across groups when it comes to interactions with social institutions and personal safety.

As discussed previously, one of the most comprehensive recent discussions of policy options to address discrimination in America was raised by the 2014 report, *Falling Further Behind*.^1^ In particular, for the area of health care, the report focused on a landmark Affordable Care Act provision (Section 1557) prohibiting discrimination in almost all areas of the health care system on the bases of race, color, national origin, sex, age, or disability. This represents the first federal law containing a broad prohibition on sex discrimination in health programs, and these types of laws have the potential to be powerful tools for addressing discrimination in the future. However, health services researchers should take note of major recent changes in federal policies of this type that limit federal and state action to end discrimination. For example, in May 2019, the Department of Health and Human Services proposed a new rule dramatically reversing the agency's prior broad interpretation of Section 1557, eliminating major sections of the rule as originally written.^16^ Future work should study the effects and effectiveness of how the ACA and other health care laws have impacted the incidence of discrimination in health care and health disparities that result. This research should be conducted under different social and political conditions, particularly for groups that have been overlooked in prior research.

The issues covered by this polling effort involve over a dozen aspects of American life. Each of these issues carries its own ongoing debate about policies and programs that might improve life for racial/ethnic minorities, women, and LGBTQ adults by narrowing disparities and ending discrimination. However, achieving equity in areas as diverse as the workforce, policing, and voting, are extremely complex, and there is no national consensus on how these problems should be solved. Even in health care, although there is a strong body of evidence establishing the need to narrow racial and ethnic disparities,^17^ there is not a consensus on how to broadly end discrimination, which would greatly reduce these disparities. Promisingly, in response to these issues, experts have recommended a myriad of different solutions that are worthy of serious consideration and study, including policies to improve socioeconomic opportunities for minority and marginalized populations, civil and human rights protections that do not exist or are not enforced in current US law, and the inclusion of ending discrimination as an explicit goal in research, policies, and programs.^1^, ^17^, ^18^, ^19^ Our data suggest that more action is needed to reduce and eventually end discrimination in America, as most interventions aimed to discrimination reduction have not been rigorously evaluated for their effects on improving health outcomes or reducing health disparities. While it is beyond the scope of these results to make specific recommendations for how to end discrimination in each area of American life studied in this polling effort, this Special Issue provides important evidence that more research and practice on discrimination are sorely needed in health services research.

## 2 CONCLUSIONS

This Special Issue comes at a time when there is a serious national debate on whether we should move ahead with strong federal government policies aimed at reducing discrimination. The purpose of this polling effort was to extend prior research in building a base of recent evidence on reported experiences of discrimination, using national telephone polling data on groups who are often overlooked, to inform future policy debates over this issue. These findings suggest that, despite recent public perceptions to the contrary,^3^, ^6^, ^7^ reported discrimination against racial/ethnic minorities, women, and LGBTQ adults, taken from their own perspectives, is prevalent in the United States today. Given the systemic and widespread scale of these experiences of discrimination, a similarly systemic and widespread effort must be made to end these discriminatory behaviors in day‐to‐day life and in discriminatory institutions and policies. These results also present a challenge for educational institutions at all levels. Institutions need to educate not only about the pervasive nature of discrimination, but also about the harmful long‐term impacts on the country, communities, individuals, and their families if we cannot reduce discriminatory behavior and its burdens on the country today.

Discrimination has been widely shown to have significant, harmful effects on health and well‐being. Taken together, this polling effort illustrates the significant and widespread level of discrimination against many groups in America today, as well as the complex manifestation of these experiences across different groups and different areas of life. It demonstrates that, rather than isolated incidents, these experiences reflect a larger, systemic pattern of discrimination in America, with significant implications for the health, justice, and equality of both individual Americans and the future of the nation as a whole.
